# Growth status and age at peak height velocity among youth participants in several sports: the Cracow longitudinal study

**DOI:** 10.1186/s13102-024-00905-6

**Published:** 2024-05-29

**Authors:** Sławomir M. Kozieł, Agnieszka Suder, Maria Chrzanowska, Miroslav Králík, Robert M. Malina

**Affiliations:** 1 Department of Anthropology, Hirszfeld Institute of Immunology and Experimental Therapy, Polish Academy of Sciences, Rudolf Weigiel St. 12, Wrocław, 53- 114 Poland; 2 Department of Anatomy, University of Physical Education, Cracow, Poland; 3 Department of Anthropology, University of Physical Education, Cracow, Poland; 4 Department of Anthropology, Faculty of Science, Masaryk University, Brno, Czech Republic; 5 Department of Kinesiology and Health Education, School of Public Health and Information Sciences, University of Texas at Austin, University of Louisville, Louisville, KY USA

**Keywords:** Adolescence, Growth spurt, Longitudinal studies, Athletes, Sex differences, Youth sports

## Abstract

**Background:**

Studies addressing age at peak height velocity (PHV) in longitudinal samples of participants in sports are relatively limited.

**Purpose:**

To compare the growth status and estimated ages at PHV of longitudinal samples girls and boys active in sport with peers not active in sport, and to compare estimated ages at PHV among longitudinal samples of Polish youth active in sport.

**Methods:**

Records from the Cracow Longitudinal Study, which measured youth annually from 8 to 18 years, were screened to identify individuals regularly active in sport. Participants in athletics (22 girls, 10 boys), soccer (12 boys), and other team (6 girls, 7 boys) and individual (6 girls, 9 boys) sports were identified; 107 girls and 172 boys were not active in sport. Heights and weights of participants in sports and non-involved peers were compared. Longitudinal height records of individuals were fit with the SITAR model to estimate age at PHV. Ages at PHV of boys and girls active in sport and peers not active in sports were compared with sex-specific ANOVAs.

**Results:**

Ages at PHV of boys participating in athletics and soccer were similar to age at PHV of boys not active in sport, while ages at PHV of boys in other team sports (basketball, volleyball, handball) and individual sports (skiing, gymnastics, acrobatics) were, respectively, slightly earlier and later. Among girls, age at PHV of participants in team sports (basketball, netball) was earlier, while ages at PHV among participants in athletics and other individual sports (equestrian, acrobatics, shooting) were slightly later compared to non-athlete peers.

**Conclusion:**

Ages at PHV varied among participants in different sports and were consistent with estimates in other longitudinal samples of Polish youth athletes.

**Supplementary Information:**

The online version contains supplementary material available at 10.1186/s13102-024-00905-6.

## Introduction

Studies of youth athletes often focus on the interval of the adolescent growth spurt, which starts in late childhood and continues through the teen years, and includes sexual maturation and marked increments of growth in height and weight, corresponding changes in body composition, physiological functions, strength and motor performance [1–7]. Age at peak height velocity (PHV), the estimated age at the maximum rate of growth in height, and the estimated peak velocity of growth in height (cm/year) are often the focus of study. Estimates of ages at PHV have a long tradition in longitudinal growth studies of the general population [8], while corresponding studies of ages at PHV among participants in sports are relatively limited [5, 9].

The purpose of this study is twofold: first, to compare the growth status and estimated ages at PHV of boys and girls in the Cracow Longitudinal Study who were active in several sports with peers who were not active in sport during the interval of adolescence, and second, to compare estimated ages at PHV in the Cracow samples of youth active in sport with ages at PHV reported for longitudinal samples of Polish youth involved in different sports. Given available data addressing ages at PHV in longitudinal samples of youth sport participants [5, 9], it was hypothesized that estimated ages at PHV will vary among participants in different sports and will also vary relative to ages at PHV among youth who do not participate in sport.

## Materials and methods

### Participants

Data were extracted from the Cracow Longitudinal Study which measured the heights and weights (among other anthropometric dimensions) of school children annually from May1980 when the children were about 8 years of age through May 1990 when subjects were about 18 years of age [10]. All participants were of Polish (European) ancestry and were enrolled in several schools within the district of Nowa Huta in the city of Cracow, southeastern Poland. The study was conducted according to the scientific and ethical principles/standards of the Helsinki Declaration, and was approved and funded by the University of Physical Education in Cracow. Verbal informed consent was obtained from the parents of participants. The use of verbal/oral informed consent was also approved by University of Physical Education in Cracow; of relevance, written consent was not required in Poland at the time of the study.

Although children and respective families were resident in Cracow at the time of the study, about 70% of parents were born in rural areas and small towns, and 80% were of from a working-class and peasant background. About 40% of parents had a basic vocational education and 23% had a secondary technical education. Most families of the participants in the Cracow study had two children [11].

### Measurements

Heights (0.1 cm) and weights (0.1 kg) were measured annually in the month of May (i.e., towards the end of each school year), beginning with children in the first grade in 1980 when children had a chronological age (CA) of about 8 years and concluding in1990 when the students had a CA of about 18 years. Measurements of height and weight (and other anthropometric dimensions) were taken by experienced staff of the Departments of Anthropology and Anatomy of the University of Physical Education in Cracow following established guidelines [10]; details of the protocols and measurement variability have been previously reported [11]. There were no differences in a cross-sectional comparison of the heights and weights of children in the longitudinal series and others resident throughout Cracow in 1983 [12]. The overall characteristics of the sample have been previously reported [12, 13].

### Longitudinal sample

The initial sample in 1980 included 460 boys and 360 girls; in 1990, 266 boys (41%) and 147 girls (58%) had reasonably complete data for height and weight. Those who dropped out during the course of the study did not differ in CA, height and weight at baseline from those who persisted. Height records spanning childhood through adolescence were available for 210 boys and 141 girls. Mean CAs at initial and final observations were identical in boys and girls, 8.0 ± 0.3 years and 18.0 ± 0.3 years, respectively.

### Sport background

During the course of the study, participants were asked to complete questionnaires dealing with school and recreational activities, including participation in sport clubs. The records included the frequency (times per week), average duration of sessions (hours), and specific activity in the sport clubs [10]. The records were subsequently evaluated to identify youth who regularly participated in a specific sport or in several sports. Of the total sample of 210 boys for whom ages at PHV were successfully estimated, 38 were regularly active in sport: 12 in soccer, 7 in other team sports (basketball, volleyball and/or handball), 10 in athletics, and 9 in other individual sports (skiing, gymnastics, acrobatics); 172 boys were not active in sport. Boys involved in soccer were treated separately from those in basketball, volleyball and handball as available data indicate significant differences in height and weight between soccer players and participants in the latter sports [14]. Of the total sample of 141 girls for whom ages at PHV were successfully estimated, 34 were regularly active in sport: 22 in athletics, 6 in team sports (5 in basketball and netball, 1 in soccer), and 6 in other individual sports (equestrian, acrobatics, shooting), while 107 girls were not active in sport.

Weekly time (means ± standard errors) of active participation in each sport group among boys was as follows: soccer, 4.2 ± 0.6 h, other team sports, 4.4 ± 0.9 h, athletics, 3.2 ± 0.5 h and other individual sports, 4.6 ± 1.0 h. Corresponding weekly duration among girls was as follows: team sports, 4.8 ± 1.2 h, athletics, 4.3 ± 0.5 h, and other individual sports, 3.4 ± 0.4 h. Weekly durations of active participation in the respective sports were generally similar among boys and girls.

Boys and girls participating in athletics were involved in running and jumping events, though specific distances (e.g., sprints, middle and distance runs) or jumps (standing long jump, high jump) were not specified. Most of the boys participating in athletics, soccer and other team sports were involved in the respective sports since 1983–1984, while those in the other individual sports were involved since 1984–1985. Among girls participating in athletics, one was involved in the sport since 1982, 7 since 1983–1984 and 11 since 1985, while the number of girls involved in team and other individual sports were involved since 1983–1984.

The majority of youth in the Cracow Longitudinal Study also participated in bicycling (typically for transport to school) and swimming (at school) during the course of the academic year. Among the 38 boys regularly active in sport, only one did not participate in bicycling and three did not participate in swimming, while among the 172 boys not active in sport, 13 did not participate in bicycling and 40 swimming. Among the 34 girls active in sport, six did not participate in bicycling and only three did not participate in swimming, while among the 96 girls not active in sport, 20 did not participate in bicycling and 33 in swimming (information was lacking for 10 girls). Skiing was also a common recreational winter activity for the majority of boys, 59% of those not active in sport (101 of 172) and 63% of those active in sport (24 of 38). In contrast, relatively few girls participated in skiing, 35% of those not active in sport (34 of 96) and only 21% of those active in sport (7 of 34).

### Comparative ages at PHV among polish youth

In the context of the second purpose of the study, ages at PHV of youth active in sport were available for participants in the Wrocław Growth Study and the Wrocław Longitudinal Twin Study [15, 16] and for participants in sport schools and clubs in Warsaw [16–19]. For the Wrocław series, athletes were identified in a similar manner as in the present study, i.e., girls and boys active in sports during childhood and adolescence were identified from longitudinal records for individuals. Accordingly, 13 girls participated in basketball, athletics and swimming, and several participated in multiple sports, while 25 boys participated in soccer, volleyball, basketball, athletics, swimming, karate and judo, and several participated in both team and individual sports.

The Warsaw series included participants in sport schools or clubs for athletics (12 girls, 10 boys) and rowing (11 girls, 11 boys) between about 11 years through adolescence [17, 18], and artistic gymnastics (9 girls, 15 boys) spanning 10–18 years [19]. The longitudinal data for youth participating in athletics and rowing were provided to RMM by Dr. Barbara Wojnarowska of the Institute of Mother and Child in Warsaw, Poland, while the data for individual gymnasts were included in the original report [19]. The data for individual athletes were modeled with several different protocols to estimate ages at PHV. Although a variety of methods is presently available to mathematically model or fit longitudinal records of height for individual youth, no single model is the standard; all have underlying assumptions and associated limitations [14].

### Statistical analysis

The longitudinal height records of individual boys and girls whose height records spanned childhood and adolescence were fit with the SITAR model [20–23] to estimate age at PHV [14]. The goodness of fit of the SITAR model had a residual variance of 1.26 cm^2^ for boys (standard error of estimate of 1.12 cm) and 0.48 cm^2^ for girls (standard error of 0.69 cm).

The heights and weights of boys and girls active and not active in sport were compared with sex-specific two-way analyses of variance, where age and sport groups were factors and height and weight were dependent variables. Estimated ages at PHV among those active and not active in sport were evaluated with sex-specific analyses of variance. Differences in particular groups were assessed by post hoc comparisons (Tukey’s HSD test). Statistical significance was set at *p* < 0.05.

## Results

### Heights and Weights

Results of the sex-specific the two-way analyses of variance, where CA groups and participants in different sports and non-participants were factors and height and weight were dependent variables are summarized in Table 1. Over the age range 8 through18 years, heights and weights differ significantly by CA and among participants in different sports and non-sport participants. Boys participating in other teams sports, athletics and other individual sports differ significantly in mean heights relative to boys not involved in sport (*p* < 0.001), while participants in soccer did not differ in height from boys not involved in sport. On the other hand, only boys participating in team sports differ significantly in body weight from those not involved in sport (*p* < 0.001). Girls participating in athletics (*p* < 0.001) and team sports differ significantly in height relative to girls not active in sport (*p* < 0.05), while girls participating in other individual sports did not differ in height from those no involved in sport. Only girls participating in team sports differ significantly in weight relative to girls not active in sport (*p* < 0.05).

Scrutinized differences within age groups showed that boys and girls not active in sport did not differ in mean height and weight from their age peers active in sport. However, within age groups from 11 to 14 years for height and 8 to 17 years for weight some sport discipline groups showed significant differences from other sport discipline group, but respective pairs of sport discipline groups which differed varied by age groups.

**Table 1:** Results of sex-specific two-way analyses of variance comparing heights and weights (dependent variables) of boys and girls (8–18 years) active in different sports and not active in sport where chronological age (CA) groups and sport discipline (including not active in sport) groups were factors

	Height		Weight	
	F	p	F	p
Boys: CA	532.0	< 0.001	266.9	< 0.001
Discipline	7.1	< 0.001	8.1	< 0.001
Interaction	0.1	ns	0.4	ns
Girls: CA	193.3	< 0.001	117.8	< 0.001
Discipline	8.9	< 0.001	7.2	< 0.001
Interaction	0.3	ns	0.4	ns

**Fig. 1:**
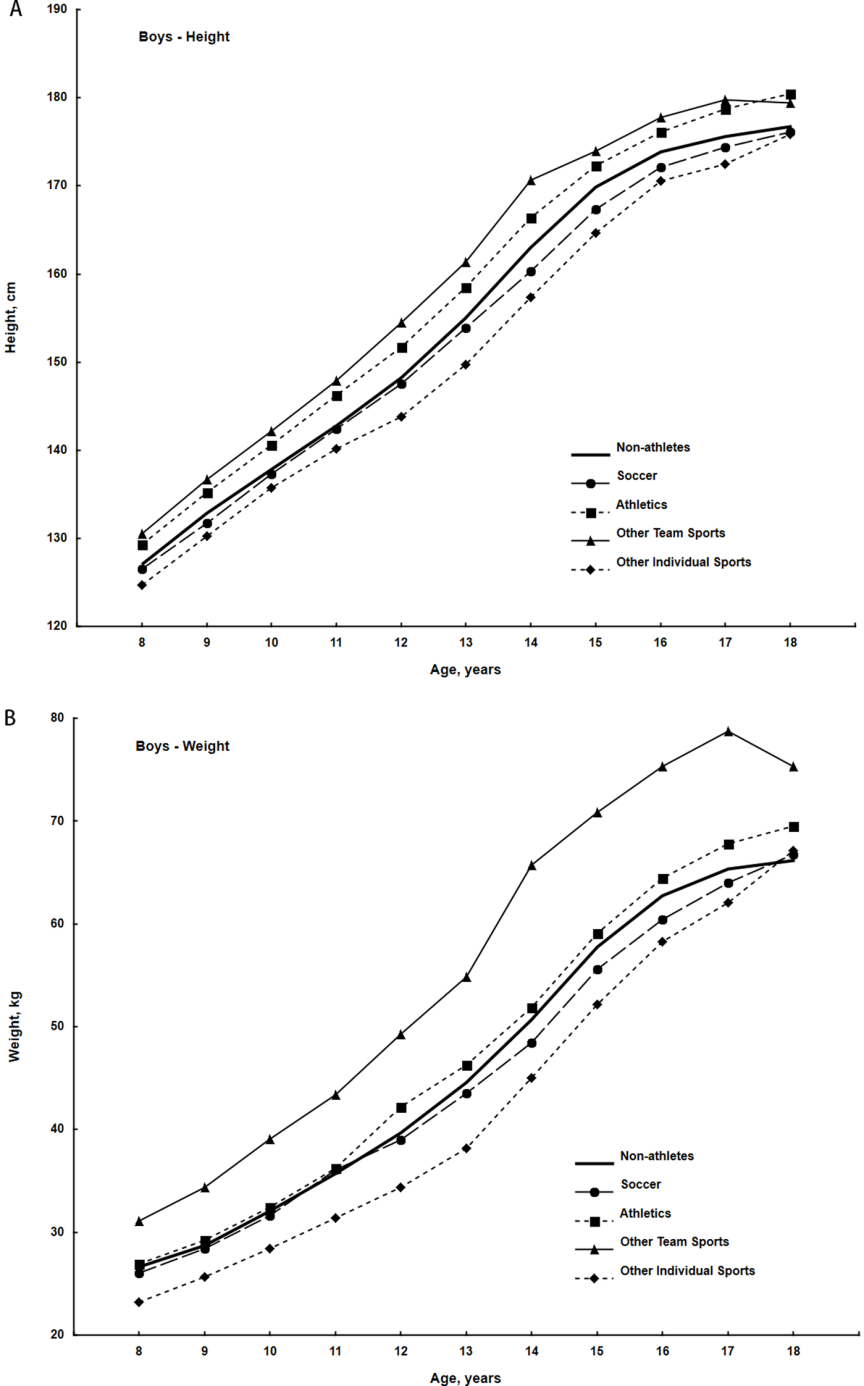
Mean heights (**A**) and weights (**B**) of non-participants in sport and of participants in several sports among boys in the Cracow Longitudinal Study

The preceding statistical analyses compared the heights and weights of participants in different sports and non-participants across the entire CA range of the respective samples. On the other hand, the plots of the heights and weights by CA groups within each sport and the sample of non-participants highlight the variation among the participants in different sports across late childhood through adolescence in the respective samples. For example, boys involved in other team sports and in athletics are, on average, taller, while those participating in soccer and in several individual sports (other than athletics) are, on average, slightly but consistently shorter than boys not active in sport (Fig. 1A). At 18 years, however, differences in heights between non-athletes and boys active in soccer and other individual sports are negligible, and all are shorter than participants in other team sports and athletics. Boys active in other team sports are also consistently heavier across the age range, while weights of boys participating in athletics and soccer are, on average, similar to those of boys not active in sport. In contrast, boys active in other individual sports are, on average, consistently lighter than boys not active in sport across the age range except at 18 years (Fig. 1B).

**Fig. 2:**
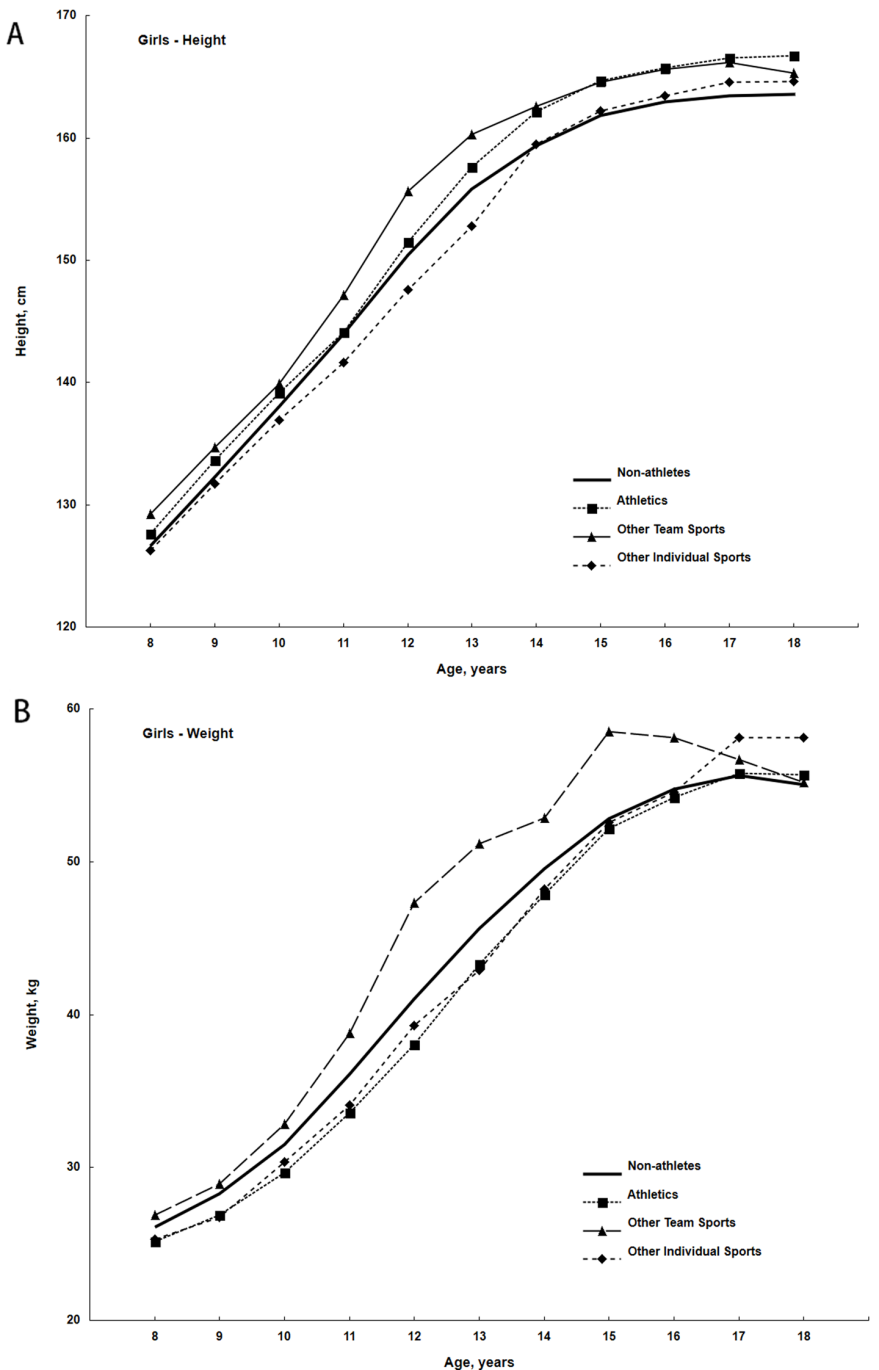
Mean heights (**A**) and weights (**B**) of non-participants in sport and of participants in several sports among girls in the Cracow Longitudinal Study

Differences in mean heights among girls in the respective groups from 8 to 10 years are small, although girls active in team sports and athletics tend to be slightly taller (Fig. 2A). Subsequently, heights of girls in the four samples diverge during adolescence. Girls active in team sports are taller than girls not active in sport from 11 to 17 years, while girls active in athletics are slightly taller than girls not active in sport from 12 years but the differences increase with age through 17 years. In contrast, girls active in other individual sports are, on average, slightly shorter than girls not active in sport through 13 years; subsequently, heights of girls in the two groups are similar.

Although differences in body weight are relatively small among the four groups of girls between 8 and 10 years, girls participating in team sports are subsequently heavier, on average, through the age range except at 17 and 18 years (Fig. 2B). In contrast, girls participating in athletics and other individual sports do not differ in weight, but are consistently lighter than non-athletes through 14 years; differences in weight are negligible among the groups at 15 and 16 years. Differences in body weight at 17 years are also negligible, though participants in other individual sports are slightly heavier.

### Ages at PHV

Ages at PHV of Cracow youth active in different sports and not active in sport are summarized in Table 2. Although ages at PHV do not differ significantly among boys active and not active in sport (F = 1.39, *p* = 0.25) and also among girls active and not active in sport (F = 1.71, *p* = 0.17), several trends are suggested. Among boys, mean ages at PHV of participants in athletics and soccer are similar to age at PHV of boys not active in sport, while mean age at PHV of participants in other team sports is slightly earlier and that of participants in other individual sports is slightly later. Among girls, age at PHV is also slightly earlier among participants in team sports compared to the non-athletes, while ages at PHV of participants in athletics and in other individual sports are slightly later compared to non-athletes.

**Table 2:** Means (M), standard deviations (SD) and ranges for estimated ages at peak height velocity (PHV) among youth in the cracow longitudinal study who were not active in sport and who were active in different sports during the interval of the study

	Ages at PHV, years							
	Girls				Boys			
	n	M	SD	Range	n	M	SD	Range
Not Involved	107	11.9	0.9	9.54–14.51	172	13.6	0.8	11.26–16.21
Athletics	22	12.2	0.5	10.72–13.76	10	13.6	1.1	12.57–15.74
Soccer	-	-	-		12	13.7	1.0	12.36–15.22
Team Sports	6	11.7	0.7	10.74–12.93	7	13.2	1.0	11.71–14.64
Individual Sports	6	12.5	0.7	11.29–13.25	9	14.1	0.7	13.08–15.23

### Comparative ages at PHV among polish youth active in sport

Estimated ages at PHV of Cracow youth participating in different sports are consistent with observations of youth athletes in Warsaw and Wrocław (Table 3). As noted earlier, several different methods of modeling longitudinal height data to estimate ages at PHV were used in the Warsaw samples of participants in athletics and rowing [17, 18] and in artistic gymnasts [19]. Allowing for variation among methods, estimated ages at PHV are generally consistent among sports except for later mean ages at PHV in artistic gymnasts of both sexes.

**Table 3:** Sample sizes, means (M) and standard deviations (SD) for estimated ages at peak height velocity (PHV) among female and male athletes in the present study and in other longitudinal studies of youth athletes in Poland

			Ages at PHV, years					
			Females			Males		
Sports	Study	Method*	n	M	SD	n	M	SD
Athletics	Cracow, this study	SITAR	22	12.2	0.8	10	13.6	1.1
Athletics	Warsaw [21]**	KR SITAR FDA	12	12.1 12.4	0.9 0.7	10	13.6 13.7 13.6	0.8 0.7 0.7
Gymnastics	Warsaw [23]**	Graphic KR Polynomials SITAR	9	13.2 13.1 13.1 13.2	1.0 0.9 0.8 0.7	15	14.9 14.7 14.8 14.8	1.0 1.1 1.1 0.9
Rowing	Warsaw [21]**	KR SITAR FDA	11	11.8 12.3	0.9 1.0	11	12.6 12.9 12.7	0.9 0.7 0.8
Soccer	Cracow, this study	SITAR				12	13.7	1.0
Other team sports^a^	Cracow, this study	SITAR	6	11.7	0.7	7	13.2	1.0
Individual sports^b^	Cracow, this study	SITAR	6	12.5	0.7	9	14.1	0.7
Mixed sports^c^	Wroclaw [19]	PB1	13	12.3	0.8	25	13.6	0.9

*In addition to SITAR (see methods), KR = Kernel regression, FDA = functional development analysis, PB1 = Preece-Baines model 1

**As the data for heights of individual athletes were available to RMM (see text), ages at PHV were estimated with different methods

^a^girls - basketball, netball, one in soccer; boys - basketball, handball, volleyball

^b^girls - acrobatics, equestrian, shooting; boys - gymnastics, acrobatics, skiing, swimming

^c^girls - basketball, athletics, swimming, several sports; boys - soccer, volleyball, athletics, swimming, karate, judo (6 girls and 16 boys were also active in sport as young adults)

0.9

## Discussion

### Growth status relative to a recent polish reference sample

Since the heights and weights of Cracow boys and girls not active in sport served as the reference for evaluating the growth status of peers regularly involved in several sports, a question of relevance is the growth status of the Cracow youth not active in sport relative to a reference for Polish youth. Compared to reference medians for Polish youth in 2010 [24], mean heights of Cracow non-athlete boys in the 1980s were slightly shorter from 8 to 15 years, but differed negligibly at 16–18 years. Mean weights of Cracow non-athlete boys in the 1980s were also slightly below the Polish reference medians from 9 to 14 years, but at other ages approximated the reference. Mean heights of Cracow non-athlete girls in the 1980s were slightly below the 2010 Polish reference medians [24] from 8 to 15 years, but approximated the medians at 16–17 years. As in boys, mean weights of Cracow non-athlete girls in the 1980s were slightly below the Polish reference medians from 9 to 14 years, but approximated the reference medians at other ages.

The trends in mean heights and weights in the males participating in athletics and soccer were consistent with other studies of youth athletes in the respective sports [2, 25]. The taller heights and heavier weights of male participants in other team sports were also consistent with observations for basketball, volleyball and handball players, i.e., taller and heavier than average [8]. On the other hand, the small number of male participants in several individual sports (skiing, gymnastics, acrobatics) had, on average, shorter heights and lighter weights than those not involved in sport. The latter trend was generally consistent with available data for the heights and weights of gymnasts; however, limited data for skiers suggested heights and weights only slightly less than the U.S. reference median [8].

The corresponding trends in mean heights and weights of the small sample of girls participating in team sports were consistent with observations for basketball and soccer players, especially during adolescence; data for netball players are lacking [14]. The trends for heights and weights of participants in athletics were also consistent with heights and weights of girls participating in general athletics, i.e., not specialized in the sprints, middle distance or distance runs and jumping events [2]. The trend for the small sample of girls participating in several individual sports (equestrian, acrobatics, shooting) indicated shorter heights during the adolescent years, which likely reflected their later maturation; otherwise, their heights during late childhood and late adolescence approximated the reference. In contrast, body weights of the girls in individual sports were similar to participants in athletics and were less than the reference during adolescence; in late adolescence, however, the weights approximated the reference. Except for artistic gymnasts who present shorter heights and lighter weights from childhood through adolescence, comparative data for participants in the other individual sports represented in the small sample are lacking [8].

Ages at PHV among Youth not Active in Sport. The estimated ages at PHV for girls (11.9 ± 0.9 years) and boys (13.6 ± 0.8 years) in the Cracow Longitudinal study who were not active in sport were identical with those noted for the total sample of girls and boys in the study [14], and were also similar to estimates based on increments in height for Cracow girls, 11.8 ± 1.0 years, and boys, 13.6 ± 0.8 years [26]. The estimates for Cracow girls and boys were also consistent with observations in other longitudinal studies of Polish youth, allowing for different analytical protocols. Among youth in the Warsaw study not active in sport, ages at PHV were 11.8 ± 0.7 years in girls and 13.8 ± 1.3 years [16], while estimated ages at PHV in the Wrocław Longitudinal Study were 11.7 ± 0.9 and 11.9 ± 1.0 years in girls and 13.9 ± 1.1 and 14.1 ± 1.1 years in boys [27–30]. Estimates of ages at PHV in the Poznań Longitudinal Study were 11.8 ± 0.9 years in girls and 13.9 ± 0.8 years in boys [31], while a more recent estimate for Poznań girls was 11.5 ± 0.8 years [32]. Estimated means for a longitudinal series from Lublin were 11.3 years in girls and 13.6 years in boys; standard deviations were not reported [33, 34].

### Ages at PHV among youth in different sports

Estimated ages at PHV in the samples of Cracow youth participating in different sports were also consistent with those noted in the longitudinal samples of youth athletes in Warsaw and Wrocław, except for the later ages at PHV among youth of both sexes participating in artistic gymnastics (Table 3). Unfortunately, the current study included only one boy who participated in artistic gymnastics.

Among participants in athletics in the Cracow study, mean age at PHV for females was earlier, while that for males was later compared to ages at PHV for six female (12.6 years) and five male (13.0 years) Spanish sprinters and middle distance and distance runners [35]. In contrast, ages at PHV of both female and male Cracow participants in athletics were later than estimates for larger samples of Belgian sprinters (60 m to 400 m), 11.6 ± 1.5 years in females and 13.1 ± 1.0 years in males [36]. The estimates for Polish female rowers were similar to those for female participants in athletics, while estimates for male rowers were earlier than those for male participants in athletics.

The estimated age at PHV for the sample of Cracow soccer players (13.7 ± 1.0 years) was within the range of 13 estimated ages at PHV in seven samples of soccer players in Europe, 13.4 ± 0.8 to 14.2 ± 0.8 years [37]. Ages at PHV for the small samples of girls and boys in mixed team sports (other than soccer among boys) were earlier, while those for girls and boys in several individual sports were slightly later compared to ages at PHV in girls and boys in the Wrocław studies who participated in both team and individual sports, 12.3 ± 0.8 years and 13.6 ± 0.9 years, respectively (Table 3). On the other hand, when the samples of participants in mixed team and individual sports in the present study are combined, means ages at PHV, 12.1 ± 0.8 years in females and 13.7 ± 0.9 years in males, were similar to the Wrocław samples. Two longitudinal studies of boys in the former Czechoslovakia reported generally similar estimated ages at PHV for a sample of 8 athletes (basketball 7, running 1) in Prague, 14.1 ± 1.0 years [38] and for 17 athletes in several team and individual sports in Brno, 13.9 ± 1.2 years [39].

Some of the variation in estimated ages at PHV among athletes in different sports may reflect the different methods used to model longitudinal data in the respective studies. The SITAR model was used in the present analysis. Among boys and girls in the longitudinal samples in the Wroclaw studies, ages at PHV estimated with Preece-Baines model 1 and the SITAR model were similar. The mean differences in ages at PHV estimated with the two methods were not significant, 0.15 year in boys and 0.12 year in girls. Moreover, correlations between ages at PHV based on the two methods were high, 0.97 in boys and 0.94 in girls (unpublished data). Similarly, in a sample of male soccer players, the mean difference between ages at PHV with the SITAR and FDA methods was 0.04 year and the correlation between ages at PHV was 0.95 [37].

### Limitations

Potential limitations of the present study should be noted. The Cracow Longitudinal Study was a prospective cohort study and non-interventional. The sample was also influenced by drop-outs associated with secondary education, i.e., specifically the transfer of boys and girls from primary schools to different secondary schools at about 16 years of age. Participation in sport was self-reported via questionnaires. Sample sizes for some of the team and individual sports groups were relatively small and given the limited numbers, youth participating in different sports were combined. This could result in groups heterogeneous relative to selection, popularity and preferences for particular sport disciplines. Nevertheless, the estimated ages at PHV for Cracow youth participants in sport were consistent with a similar retrospective study of participants in two Wrocław longitudinal studies and in studies of Polish youth participating in athletics, rowing and artistic gymnastics (Table 3). Allowing for relatively small samples in some sports, the mean ages at PHV for the samples of participants in different sports were also consistent with reported mean ages at PHV in samples of youth athletes in Europe [8, 9].

It should also be noted that the focus of the study was attained body size and age at PHV among youth participants in several sports and non-participants. The present study and many longitudinal studies of youth athletes do not consider variation in the timing of the onset of the interval of the adolescence, commonly labeled as the age at take-off of the growth spurt, which is of interest to those working with youth athletes.

### Practical implications

Variation in ages at PHV among youth participating in different sports merits attention as the interval of adolescence is often viewed as a time of enhanced responsiveness to sport training – metabolic, cardiovascular and neuromuscular [40, 41]; it is also the interval when decisions regarding selection or retention are often made [5]. Although it is not always clear whether focus is the onset of the growth spurt (age at take-off), the interval between onset and PHV, or estimated age at PHV per se, focus is often on the latter. And as noted in Table 2, inter-individual variation in estimated ages at PHV within sports is considerable and should not be overlooked. Allowing for different statistical models, similar ranges of variability in ages at PHV were noted among participants in athletics, females 11.1 to 13.7 years and males12.9 to 15.1 years; rowing, females 10.9 to 14.3 years and males 11.2 to 14.2 years; and artistic gymnastics, females 12.0 to 14.5 years and males 12.2 to 16.7 years [16–19, 24]. From a practical perspective, it is important that those working with youth athletes are aware of the broad range of variability among youth in the timing of the growth spurt.

In contrast to estimated ages at PHV in longitudinal samples of youth active in sport, cross-sectional studies of athletes increasingly use predicted maturity offset [42, 43], defined as the time before and/or after PHV, as a maturity indicator. Age at PHV is estimated as the difference between CA at the time of observation and predicted maturity offset. Applications of the prediction equations to several longitudinal series for which ages at PHV for individual youth were estimated have noted several limitations. Predicted ages at PHV based on maturity offset varied with CA and height at prediction, and had a reduced range of variability relative to observed ages at PHV. Moreover, predicted ages at PHV had major limitations among early and late maturing youth defined by observed ages at PHV [14, 30, 44–46]. Similar results have been noted in longitudinal samples of female gymnasts [47] and male soccer players [48–50]. Given the consistent results in the longitudinal samples of non-athletes and athletes, those working with youth athletes should be aware of the limitations of the maturity offset prediction equations as well as variability in measurements of height and sitting height which are among the predictors [42].

The issue of injury risk during the interval of the adolescent spurt also merits attention [51]. In a longitudinal sample male soccer players at an elite club, for example, injuries recorded over time were considered relative to estimated ages at PHV for individual players [52]. Of interest, days lost per player/season associated with growth-related injuries, labeled the burden of injury, was greatest during the interval of PHV and lowest during the interval before PHV. On the other hand, the burden associated with muscle and joint/ligament injuries was higher among post-PHV and lowest in pre-PHV players.

Although interesting, the association of injury with the interval of PHV does not imply a cause-effect sequence. There is a need to also consider the growth and functional characteristics of players and the contexts of injuries, e.g., practice or game, specific activities during practice, game situations, coach demands and pressures, and so on. Nevertheless, the results highlight the importance of the interval of the adolescent spurt in studies of youth athletes and the need for awareness individual differences in the timing and tempo of this maturational event and of course variation in methods of assessment.

A question of potential relevance that often surfaces in discussions of youth athletes is the following: Were youngsters involved in a specific sport because they were later maturing, or was the later maturation associated with systematic training in a sport? This is a difficult question to answer given variation in selection practices and training environments of different sports and clubs which influence whether a youngster persists in or drops out of a sport. On the other hand, available data based on short and long term longitudinal studies of youth athletes in several sports indicate no influence of regular training for sport on indicators of maturity status, timing and tempo [53]. Similarly, regular training for sport does not influence growth in stature but may influence body weight, specifically body composition [53].

## Summary and conclusion

The hypothesis of the study that growth status differs and estimated ages at PHV vary among participants in different sports and also vary relative to youth who do not participate in sport was generally supported by the results. Heights and weights differed significantly by CA and among participants in different sports and non-sport participants. Youth active in sport, except boys participating in soccer, and except girls participating in other individual sports, differed significantly in height relative to peer not active in sport. On the other hand, only girls participating in team sports differed significantly in weight relative to girls not active in sport.

Estimated ages at PHV of boys participating in athletics and soccer were similar to age at PHV of boys not active in sport, while ages at PHV of boys in other team sports (basketball, volleyball, handball) and in other individual sports (skiing, gymnastics, acrobatics) were, respectively, slightly earlier and later. Among girls, age at PHV of participants in team sports (primarily basketball, netball) was earlier, while ages at PHV among participants in athletics and other individual sports (equestrian, acrobatics, shooting) were slightly later compared to non-athlete peers. Estimated ages at PHV of youth from the Cracow Longitudinal Study who participated in several sports were also consistent with corresponding estimates based on other longitudinal studies of Polish youth active in several sports.

### Electronic supplementary material

Below is the link to the electronic supplementary material.

Supplementary Material 1

## Data Availability

The data that support the findings of this study are not publicly available due to departmental policy and privacy commitments to the study participants. Nevertheless, the data may be available upon reasonable request to Agnieszka Suder, University of Physical Education, Cracow, Poland (agnieszka.suder@awf.krakow.pl).
