# Supplementary material for: Growth status and age at peak height velocity among youth participants in several sports: the Cracow longitudinal study
Source: BMC Sports Sci Med Rehabil. 2024 May 29;16:121. doi: 10.1186/s13102-024-00905-6 (PMC11134692; doi:10.1186/s13102-024-00905-6)
Supplement: Supplementary file 1 — Supplementary Material 1 [file 13102_2024_905_MOESM1_ESM.pdf]

Questions used in the original questionnaire

1. Father’s date of birth

2. Place of father’s birth

3. Father’s social origin

4. Level of father’s education

5. Father’s profession

6. Father’s position in work

7. Year of father arriving to Nowa Huta

8. From where did father arrive

9. Did father do sport?

10. Did father belong to a sports club?

11. Height of father (declared)

12. Weight of father (declared)

13. Mother’s date of birth

14. Place of mother’s birth

15. Mother’s social origin

16. Level of mother’s education

17. Mother’s profession

18. Mother’s position in work

19. Year of mother arriving to Nowa Huta

20. From where did father arrive

21. Did mother do sport?

22. Did mother belong to a sports club?

23. Height of mother (declared)

24. Weight of mother (declared)

25. Number of children in family

26. How many still are alive

27. How many are working professionally

28. Birth order

29. How many people contribute to running a house

30. Does family possess: flat, washing machine, phone, car

31. How long have parents been together

32. Results of examination of posture defects

33. Blood groups of father ABO and Rh

34. Blood groups of mother ABO and Rh

35. Blood groups of child (examined) ABO and Rh

36. Birth weight

38. Birth length

39. Kind of delivery

40. Gestational time

41. APGAR points

42. Weight after 1 year

43. Kind and time of breastfeeding

44. Information about breakfasts (I and II), lunch, tea, dinner

45. Activity during holiday: sport camp, with family, holiday in countryside in family, youth holiday camp, did not leave a city

46. Information about free time: homework, reading, TV, cinema, Theatre, trips, sports

47. Does the child belong to a sports club? (yes/no)

48. Provide the date (year) when your child joined the sports club:

49. Name the sport your child practices:

50. Enter the number of training hours (duration) per week (frequency):

51. Results of measures of height and weight of parents.

52. Information about completeness of family

53. Information about comfort of leaving

54. Information about conflicts in family
